# Effects of *Curcuma longa* L. and Green Propolis Extract-Loaded Microcapsules Supplementation on Inflammation in Hemodialysis Patients: Preliminary Results of a Randomized Clinical Trial

**DOI:** 10.3390/life15060891

**Published:** 2025-05-30

**Authors:** Isadora Britto, Heloiza Couto, Bruna Regis de Paiva, Jessyca S. de Brito, Livia Alvarenga, Ludmila F. M. F. Cardozo, Paulo Emilio Correa Leite, Andresa A. Berretta, Marcelo Ribeiro-Alves, Virgílio Pimentel Delgado, Danielle Figueiredo da Cunha, Carmen Lucia Sanz, Lia S. Nakao, Denise Mafra

**Affiliations:** 1 Graduate Program in Biological Sciences—Physiology, Federal University of Rio de Janeiro (UFRJ), Rio de Janeiro 21941-902, RJ, Brazil; isadorabkopke@gmail.com (I.B.); jessyca.sousa.brito@gmail.com (J.S.d.B.); 2 Graduate Program in Nutrition Sciences, Fluminense Federal University (UFF), Niterói 24030-215, RJ, Brazil; heloizanutri@hotmail.com (H.C.); bruna.regis.paiva@gmail.com (B.R.d.P.); liviaalvarenga92@gmail.com (L.A.); ludmila.cardozo@gmail.com (L.F.M.F.C.); 3 Renal Medical Assistance, Teresópolis 25975-450, RJ, Brazil; virgiliodelgado@gmail.com (V.P.D.); daniellemedicina@hotmail.com (D.F.d.C.); 4 Graduate Program in Cardiovascular Sciences, Fluminense Federal University (UFF), Niterói 24030-215, RJ, Brazil; 5 Graduate Program in Science and Biotechnology, Fluminense Federal University (UFF), Niterói 24030-215, RJ, Brazil; leitepec@gmail.com; 6 Research, Development & Innovation Department, Apis Flora Industrial e Comercial Ltda, Ribeirão Preto 14020-670, SP, Brazil; andresa.berretta@apisflora.com.br; 7 HIV/AIDS Clinical Research Center, National Institute of Infectious Diseases Evandro Chagas (INI/Fiocruz), Rio de Janeiro 21040-360, RJ, Brazil; mribalves@gmail.com; 8 Department of Basic Pathology, Federal University of Paraná, Curitiba 81531-980, PR, Brazil; lusanz.alarta@gmail.com (C.L.S.); lia.nakao@ufpr.br (L.S.N.); 9 Graduate Program in Medical Sciences, Fluminense Federal University (UFF), Niterói 24030-215, RJ, Brazil

**Keywords:** chronic kidney disease, hemodialysis, inflammation, propolis, turmeric, *Curcuma longa* L., microcapsules, nutrition

## Abstract

Foods such as *Curcuma longa* L. and propolis can attenuate inflammation in patients with chronic kidney disease (CKD) undergoing hemodialysis (HD). This study aimed to evaluate the effects of microcapsules loaded with *Curcuma longa *L. and propolis on inflammatory markers and uremic toxins in patients undergoing HD. In this randomized, double-blind clinical trial, 40 patients were divided into two groups: an intervention group (137 mg/day of Curcuma and 500 mg/day of green propolis) in the form of microcapsules, and a placebo group, both administered for 8 weeks. Cytokines were analyzed using a multiplex assay (Bio-Plex Magpix^®^). Malondialdehyde was evaluated as a marker of lipid peroxidation. Uremic toxins were analyzed by reversed-phase high-performance liquid chromatography. Demographic and clinical data were obtained from medical records. A total of 38 patients completed the study: 18 were in the intervention group (49 ± 16.2 years; 8 men) and 20 were in the control group (49 ± 18.7 years; 10 men). There was a reduction in levels of C-reactive protein (*p* = 0.026) and MIP-1 (*p* = 0.019) in the intervention group. No change in uremic toxins was observed. In conclusion, the intervention with microcapsules containing *Curcuma longa* L. and green propolis showed potential anti-inflammatory effects in patients with CKD undergoing HD. These findings warrant investigation in larger, long-term trials.

## 1. Introduction

Patients with chronic kidney disease (CKD) exhibit a chronic and exacerbated systemic inflammatory state with a multifactorial origin, including gut dysbiosis and oxidative stress [1,2,3]. Oxidative stress, defined as an imbalance between the production of reactive oxygen species (ROS) and the body’s antioxidant capacity, is a key driver of inflammation. It promotes the activation of nuclear factor kB (NF-κB), a transcription factor that regulates the synthesis of inflammatory cytokines, such as several interleukins (IL-1, IL-6, IL-8, IL-1β, IL-18), tumor necrosis factor-α (TNF-α), interferon-γ (INF-γ), and other molecules, such as vascular cell adhesion molecule-1 (VCAM-1) and intercellular adhesion molecule-1 (ICAM-1), all of which are closely linked to the progression of cardiovascular diseases [4,5,6].

Gut dysbiosis in patients with CKD has multiple causes, including reduced fiber intake, medication use, and damage to the intestinal barrier caused by elevated urea levels. As a result of gut dysbiosis, these patients experience an accumulation of uremic toxins, such as p-cresyl sulfate (PCS), indole-3-acetic acid (IAA), and indoxyl sulfate (IS), which are associated with increased systemic inflammation and cardiovascular mortality [7,8,9,10].

Nonpharmacological strategies, particularly the use of bioactive compounds in food, have been increasingly studied and promoted as a means to mitigate inflammation and gut dysbiosis [11,12]. Propolis and *Curcuma longa* L. are foods with antimicrobial, antioxidant, and anti-inflammatory properties, showing promising results in CKD [13,14,15,16]. Propolis is a resin produced by bees from a mixture of buds and plant exudates, including pollen, wax, and bee enzymes [17,18]. *Curcuma longa* L. is a rhizome that was initially from India and is currently cultivated worldwide [19].

The beneficial effects of both compounds can be attributed to their phenolic constituents. Propolis is rich in caffeic acid derivatives and prenylated compounds such as artepillin C [20,21]. *Curcuma longa* L. contains curcuminoids whose phenolic radicals can stabilize electrons and neutralize ROS [22]. The benefits of the bioactive compounds present in turmeric and propolis are accompanied, however, by limitations in their use, such as the fact that they have low solubility in aqueous media, rapid metabolism in the gastrointestinal tract (GIT), and a short half-life [23]; thus, the use of microcapsules is an alternative for its safe, stable, and sustained delivery [24,25,26].

Microencapsulation ensures the optimal microbiological preservation and chemical stability of bioactive compounds [27,28,29]. Its primary role is to promote protection against digestive processes and release it at a programmed time in the gastrointestinal tract for good absorption. Polymers derived from fatty acids, waxes, cellulose, gum arabic, xanthan gum, and other sources are commonly used as encapsulation materials for food products [27,29,30,31,32]. Thus, this study aimed to evaluate the effects of microcapsules containing turmeric and propolis on the inflammatory markers and uremic toxins originating from the gut microbiota of patients with CKD undergoing HD.

## 2. Materials and Methods

This study is a longitudinal, double-blind, placebo-controlled, randomized clinical trial with patients undergoing HD at Renal Medical Assistance, Teresópolis, Rio de Janeiro, Brazil, from July to November 2021. The sample calculation for determining the study population was performed using the G-Power 3.1 software, considering tumor necrosis factor-alpha (TNF-α) [33]. With a test power of 80%, a significance level of 5% (two-tailed), and an effect size of 1.32, the sample consisted of 34 patients, with 17 allocated to the placebo group and 17 to the intervention group.

### 2.1. Inclusion and Non-Inclusion Criteria

Patients with stage 5 CKD (estimated glomerular filtration rate < 15 mL/min) undergoing HD for more than six months, aged over 18 years, and who had an arteriovenous fistula (AVF) as vascular access were included in this study.

**Figure 1:**
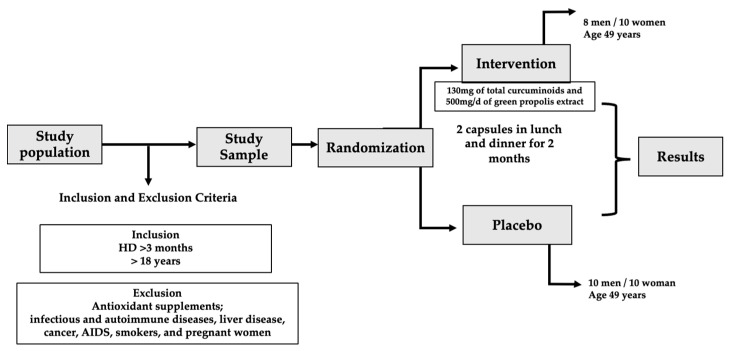
Flow chart of the included and excluded patients.

Pregnancy, antibiotic use in the last three months, use of antioxidant supplements, and who had a regular intake of prebiotic, probiotic, synbiotic, propolis, saffron, and turmeric, in addition to those with autoimmune and infectious diseases, cancer, liver diseases, and acquired human immunodeficiency syndrome (AIDS) were not included in this study (Figure 1).

### 2.2. Ethical Procedures

The project was approved by the Ethics Committee of the Hospital Universitario Pedro Ernesto-UERJ under CAAE: 52742921.1.0000.5259 and registered in the international clinical trial database ClinicalTrials.gov (NCT05183737). After obtaining informed consent, demographic, clinical, and biochemical data were collected.

### 2.3. Composition of Propolis and Curcuma longa L.

The standardized extract of green propolis (EPP-AF^®^) was used in this study and provided by Apis Flora Indl. Coml. Ltd. (Ribeirão Preto, São Paulo, Brazil). The dry extract of propolis (microcapsules) was obtained from the formulation of the emulsion prepared with the standardized hydroalcoholic extract of green propolis (EPP-AF^®^) (phase a) and gum Arabic that was previously dispersed into purified water (phase b), obtained with the dispersion of phase a into phase b with mechanical agitation and emulsion with the equivalent of 40% of total dry matter, which passed through the spray-drying system to obtain the microcapsules, as previously published by Marquiafável with modifications [34] and Berretta et al. (2023) [35]. After evaporation of the ethanol portion of the emulsion by a spray-drying system, which was formulated to achieve approximately 40% *w*/*v* dry matter of propolis in gum arabic, the extract was collected and evaluated for parameter analysis and characterization of the system. The chemical analyses were previously described by Berretta et al. (2012) [36] for determining the chemical profile using a HPLC system, quantifying the main bioactive compounds, such as artepellin C, and summing the other compounds to express the results in terms of total phenolic compounds.

The turmeric extract (*Curcuma longa* L.) was purchased from the company ActivePharmaceutica^®^. The extract was evaluated and characterized using the HPLC technique, as described in a method previously published by Jayaprakasha et al. (2002) [37]. The dry extract of turmeric in microcapsules was obtained by solubilizing the turmeric extract in alcohol (phase a), followed by the dispersion of phase a into phase b—which contains gum arabic previously dissolved in water (emulsion preparation)—in such a proportion as to offer a final powder containing about 40% *w*/*w* of turmeric. The process was performed similarly to the development of propolis microcapsules, with modifications as described by Marquiafável et al. 2015 [34]. Table 1 describes the levels of curcuminoids and propolis polyphenols in the supplement. It displays the chemical composition of the daily supplement dose in a microcapsule format.

The chromatographic profile of Curcuma longa L and propolis capsules (1) caffeic acid; (2) p-coumaric; (3) 3.5-dicaffeoylquinic acid; (4) 4,5-dicaffeoylquinic acid; (5) aromadendrin; (6) drupanin; (7) artepillin C and (8) Baccarin are available in the Supplementary Material (Figure S1).

Just like the chromatographic profile of Curcuma longa L. and propolis capsules (1) bidemethoxycurcumin; (2) demethoxycurcumin; (3) curcumin (Figure S2).

### 2.4. Experimental Design

An external researcher computerized this study’s randomization (1:1), considering the participants’ baseline characteristics such as sex, age, body mass index (BMI), adequacy of dialysis (Kt/V), and time on hemodialysis.

The dosage of supplementation consisted of ingestion of 4 daily capsules of the supplement (2 capsules after lunch and two capsules after dinner), totaling 137 mg of microencapsulated *Curcuma longa* L. (130 mg of curcuminoids) (ActivePharmaceutica^®^ Santa Catarina, Brazil) and 500 mg of microencapsulated standardized green propolis extract (EPP-AF PROPOMAX^®^, Apis Flora Industrial e Comercial Ltd.a, Ribeirão Preto, Brazil) for eight weeks. The supplementation period was defined based on previous studies evaluating either curcumin or propolis supplementation individually, which demonstrated beneficial effects on oxidative stress and inflammatory markers within timeframes ranging from 4 to 12 weeks [23,38].

The propolis and *Curcuma longa* L. microcapsule mixture was delivered as hard gelatin capsules. The placebo group was instructed to consume the same number of capsules, at the exact times, containing gum arabic and magnesium stearate, also produced by Apis Flora. The soft gels given to the placebo group were identical to those given to the intervention group in terms of all sensory characteristics.

To ensure good adherence, a calendar containing the days and times of supplementation was provided along with the supplementation. In addition, patients were contacted weekly by phone call and/or app message to encourage treatment adherence and monitor for possible adverse effects or intolerances.

The doses of propolis and *Curcuma longa* L. offered were based on the scientific literature, especially the studies by Chermut et al. (2023) [38] for the amounts of propolis and the food supplement norm of the National Health Surveillance Agency (ANVISA) for those of *Curcuma longa* L. [39].

To assess patient compliance, after the supplementation period, the remaining capsules in the bottle were requested to be returned and counted. Participants were monitored weekly in the dialysis sessions for the appearance of signs and symptoms.

### 2.5. Food Intake Analysis and BMI Measurement

Food intake was measured using a 3-day 24-h recall at the beginning and end of the intervention. The participants’ diet composition was measured according to the Brazilian Food Composition Table [40].

The BMI was calculated by dividing the body weight measured after hemodialysis by the square of height. The results were classified according to the World Health Organization [41]. Due to sanitary measures for the prevention and control of COVID-19, measurements of circumferences and skinfolds were not performed. Trained professionals performed anthropometric and biochemical measurements before and after the intervention.

### 2.6. Blood Collection

Blood samples were collected in the morning after an overnight fast of at least 8 h before the hemodialysis procedure and immediately following the arteriovenous fistula puncture. Vacutainer^®^ tubes (Becton, Dickinson and Company, Cuautitlán, Mexico) containing anticoagulant ethylenediaminetetraacetic acid (EDTA) (1.0 mg/mL) were used to obtain plasma, and Vacutainer^®^ tubes containing clot activator and separator gel were used to collect serum.

After collection, the blood was kept cold at 2 to 6 °C and transported to the Clinical Research Unit (UPC-UFF), where it was centrifuged at 2500 rpm for 10 min at 4 °C to obtain serum and plasma, which were stored in a freezer at −80 °C until analysis.

### 2.7. Inflammatory Cytokines

The multiplex assay measured inflammatory cytokines using magnetic microspheres and was performed according to the manufacturer’s recommendations, utilizing the Bio-Plex Magpix kit and device (Biorad Laboratories Inc., Hercules, CA, USA).

The following cytokines were evaluated using the following method: inflammatory macrophage protein-1β (MIP-1β/CCL-4), macrophage colony-stimulating factor (M-CSF), and interleukin-8. Interleukin 6 and tumor necrosis factor-alpha (TNF-α) were measured by the ELISA method using Peprotech brand kits (PeproTech Inc, Rocky Hill, Connecticut, United States). C-reactive protein (CRP) plasma levels were measured using the Bioclin (Quibasa—Química Básica Ltda, Belo Horizonte, Brazil) device.

### 2.8. Uremic Toxins

Plasma levels of pCS, IS, and IAA were quantified using high-performance liquid chromatography (HPLC). Briefly, 100 μL of blood plasma was diluted to a final volume of 360 μL with the addition of an internal standard (4-ethylphenol, 1.24 mM), followed by protein denaturation at 95 °C for 30 min. The samples were then cooled on ice for 10 min and centrifuged at 12,000× *g* for 20 min at 4 °C. The resulting supernatant was transferred to 30 kDa molecular weight cut-off centrifugal filters and centrifuged again at 10,000× *g* for 30 min at 4 °C. An aliquot of 10 μL of the filtrate was injected into the HPLC system and analyzed at a flow rate of 0.7 mL/min using a mobile phase consisting of methanol and 50 mM ammonium formate buffer (pH 3.0), with gradient changes adapted to the fluorescence properties of each analyte. Toxins were detected by fluorescence with the following excitation/emission wavelengths: pCS: λexc = 265 nm/λemi = 290 nm, IS: λexc = 280 nm/λemi = 383 nm, and IAA λexc = 280 nm/λemi = 383 nm. The retention times for each compound were as follows: IS: 4.8 min, pCS: 8.5 min, and IAA: 14 min. Quantification was performed by integrating the peak areas and comparing them with authentic standards using the internal standard for normalization.

### 2.9. Biochemical Parameters and Lipid Peroxidation Marker

Biochemical parameter analyses, including total cholesterol (TC), high-density lipoprotein (HDL), low-density lipoprotein (LDL), triglycerides (TG), albumin, phosphorus, calcium, urea, and creatinine, were performed using the Bioclin device.

Lipid peroxidation was estimated by the determination of reactive substances to thiobarbituric acid (TBARS), among them being malondialdehyde (MDA), using the method of Ohkawa, Ohishi, and Yagi (1979) [42].

### 2.10. Statistical Analysis

The plasma levels of the studied variables were log-transformed when indicated. Several linear mixed-effects models were used to assess the time–intervention interactions, and the patients were considered a random effect. Multiple linear fixed-effects models were used to evaluate the differences between the final (2-month) and baseline times. They were adjusted for confounding variables, including the time on HD, sex, age, BMI, medications, and the presence of comorbidities.

Continuous variables were expressed as medians and interquartile ranges (IQRs), while categorical variables were presented as percentages. The results were presented graphically for the estimated mean marginal effects and their 95% confidence intervals. All other variables in the mixed linear multiple models were kept at their mean values or in equal proportions, and contrasts were constructed from these mean marginal effects.

The Tukey Honest Significant Difference (HSD) method was used to correct the *p*-values for the number of comparisons. *p*-values < 0.05 were considered statistically significant. R software version 4.1.1 and the packages ‘lme4’, ‘emmeans’, and their dependencies were used to perform the statistical analyses.

## 3. Results

**Figure 2:**
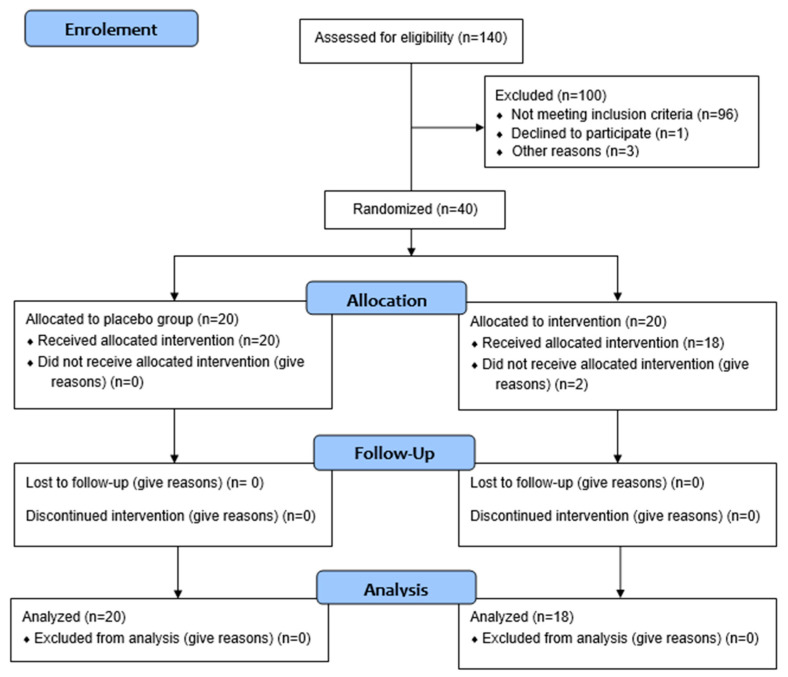
CONSORT flow diagram of the study regarding microcapsule supplementation in patients with CKD undergoing HD.

As shown in CONSORT (Figure 2), 38 patients completed the study after two months of supplementation, with 18 in the intervention group and 20 in the placebo group.

Regarding medication, 16% reported using oral hypoglycemic agents and/or insulin, 71% antihypertensive drugs, 42% iron-containing supplements, 5% hypocholesterolemic agents, and 45% phosphorus binders. All statistical analyses were adjusted to account for the use of these medications. There was no change in prescriptions during the study period.

At baseline, the two groups (placebo and microcapsules) were comparable in most demographic and clinical parameters, including sex distribution, age, dialysis vintage, and Kt/V (*p* > 0.05). However, a statistically significant difference was observed in the BMI, with the microcapsule group showing lower values compared to the placebo group (*p* = 0.034). The prevalence of comorbidities such as hypertension and diabetes did not differ significantly between groups (Table 2).

The baseline biochemical parameters were similar between the placebo and microcapsule groups, with no statistically significant differences observed in the lipid profile (total cholesterol, HDL, LDL, and triglycerides), albumin, phosphorus, calcium, urea, or creatinine levels (*p* > 0.05). Although the CRP levels were higher in the microcapsule group compared to the placebo, this difference did not reach statistical significance (*p* = 0.105) (Table 3).

Regarding the adverse effects, no significant changes in liver function or the appearance of clinical signs and symptoms were observed.). The mean compliance of patients who returned the remaining capsules was 77.0%. At baseline, no statistically significant differences were observed between the placebo and microcapsule groups in terms of energy, macronutrient, and micronutrient intake (*p* > 0.05). Both groups exhibited similarly low energy intake (~1350 kcal/day) and suboptimal calcium intake, which are common findings in patients undergoing hemodialysis (Table 4). Additionally, no significant changes were observed following the intervention.

Table 5 presents the baseline levels of inflammatory cytokines, uremic toxins, and MDA in plasma. There were no statistically significant differences between groups regarding inflammatory cytokines (IL-6, IL-8, MIP-1β, GM-CSF, and TNF-α), uremic toxins (indoxyl sulfate, p-cresyl sulfate, and indole-3-acetic acid), or the lipid peroxidation marker malondialdehyde (MDA) (*p* > 0.05 for all comparisons).

**Figure 3:**
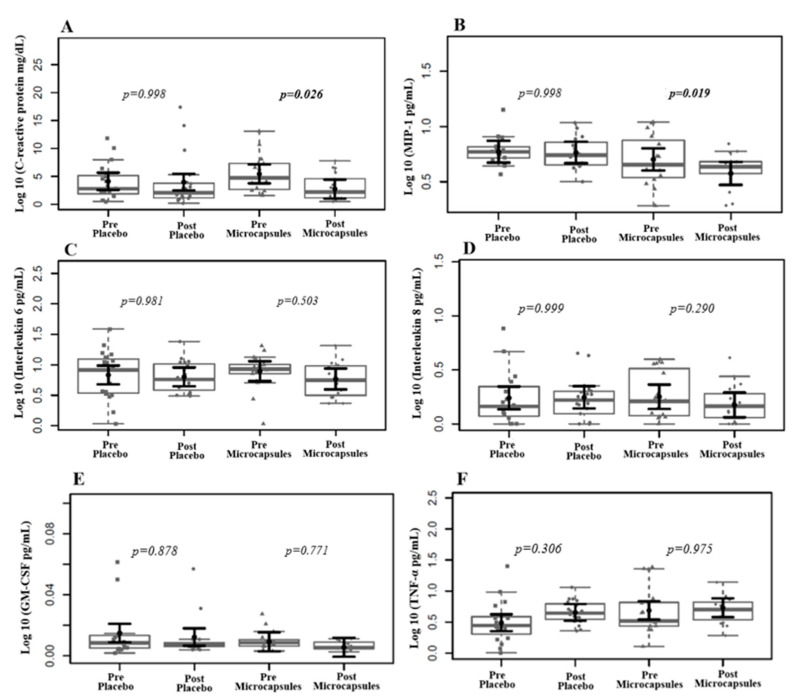
Effects of placebo supplementation and intervention on CRP and cytokine plasma levels. A significant reduction in the CRP (**A**) and MIP-1 (**B**) plasma levels was found after two months of intervention. We found no significant decrease in the levels of IL-6 (**C**), IL-8 (**D**), GM-CSF (**E**), and TNF-α (**F**) after two months of supplementation. In gray, the sampling distributions of the data are represented in the form of box plots and strip plots. In black, the center circle represents each group’s expected average marginal effect, estimated from the linear fixed-effects models. The fixed effects in the models were the intervention group, time, and their interaction, while the random effect was the patient, and the confounding effects were the time on HD and the BMI before the intervention. The black horizontal bars represent the 95% confidence intervals of the mean expected marginal effects by the group. *p*-values were corrected for the number of two-by-two contrasts/comparisons using the Tukey Honest Significant Difference (HSD) method.

**Figure 4:**
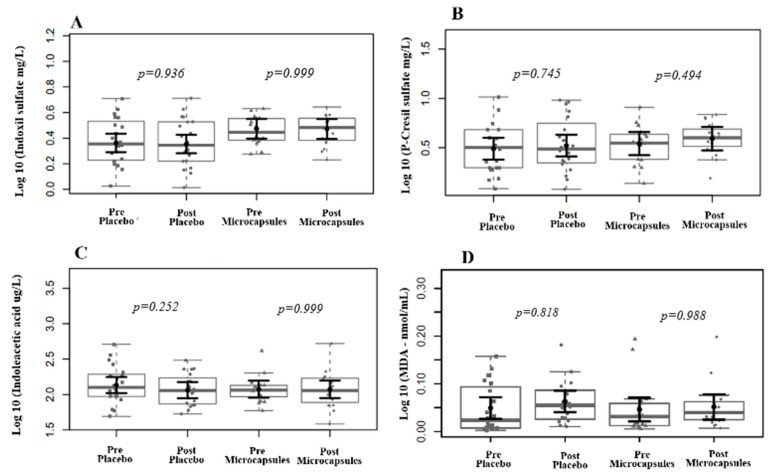
Effects of placebo supplementation and intervention on uremic toxin and MDA plasma levels. We found no significant reduction in the levels of IS (**A**), PCS (**B**), IAA (**C**), and MDA (**D**) after two months of supplementation. In gray, the sampling distributions of the data are represented in the form of box plots and strip plots. In black, the center circle represents each group’s expected average marginal effect, estimated from linear fixed-effects models. The fixed effects in the models were the intervention group, time, and their interaction. The random effect was the patient, and the confounding effects were time on HD and BMI before the intervention. The black horizontal bars represent the 95% confidence intervals of the mean expected marginal effects by the group. *p*-values were corrected for the number of two-by-two contrasts/comparisons using the Tukey Honest Significant Difference (HSD) method.

The intervention group exhibited statistically significant reductions in CRP and MIP-1 plasma levels, suggesting a potential anti-inflammatory response, although the other markers remained unchanged (Figure 3). Also, we found no change in the MDA or uremic toxin plasma levels (Figure 4).

## 4. Discussion

The preliminary results of this double-blind, randomized clinical trial suggest the potential anti-inflammatory effects of *Curcuma longa* L. (130 mg curcuminoids/day) and green propolis (500 mg/day) in patients undergoing hemodialysis, as evidenced by the reduction in selected markers. However, the absence of significant changes in several key cytokines and the short duration of the intervention underscore the need for cautious interpretations. These results should be considered preliminary and require confirmation in future studies with larger sample sizes and longer follow-up periods.

Samadian et al. (2017) [43] observed a reduction in CRP levels in patients undergoing HD after consuming 1500 mg of turmeric for three months. With a lower dose of nano-curcumin (120 mg), which underwent processing similar to turmeric microcapsules, for three months, Rasmi et al. (2020) [44] also observed a significant reduction in CRP values.

Alvarenga et al. (2020) [33] administered 2500 mg of turmeric three times a week for three months to patients undergoing HD and observed a reduction in CRP levels, demonstrating the antioxidant potential of this compound.

Chermut et al. (2023) [38] did not observe a significant difference in CRP levels in patients undergoing HD supplemented with 400 mg of propolis extract for two months. Silveira et al. (2022) [45] supplemented patients undergoing HD with 200 mg/day for one month. Despite a reduction in the numerical values of CRP, statistical significance still needs to be obtained.

In response to tissue damage, the production of CRP increases in the liver due to the stimulus from the elevated plasma levels of inflammatory cytokines, such as IL-1, IL-6, and TNF-α. By enhancing Nrf2 expression and reducing the activation of NF-kB, turmeric and propolis can reduce the cellular production of inflammatory cytokines, partly by limiting macrophage activation [46]. Furthermore, Nrf2 plays a role in downregulating Keap1 protein, which regulates the redox state and inflammation. Upon release from Keap1, Nrf2 is translocated to the nucleus, where it stimulates the transcription of antioxidant enzymes, thereby promoting a reduction in the signaling factors that regulate CRP production [46,47].

The supplementation time was a determining factor in reducing the evaluated parameters. This is because CRP is a positive acute-phase protein produced by the liver, and its transcription is regulated by signals generated by cytokines produced by monocytes, macrophages, and fibroblasts activated during inflammation, such as IL-6, IL-1b, and TNFα [48,49,50]. Future studies, which include biochemical analyses of the blood at different times of supplementation, can contribute to elucidating the exact moment when the bioactive compounds with anti-inflammatory properties of turmeric and propolis can act in reducing such cytokines and PCR.

After supplementation with microcapsules, we observed a significant reduction in the levels of macrophage inflammatory protein-1 beta (MIP-1), also known as CCL4. MIP-1β is a chemokine whose production is stimulated by the activation of the immune system against possible sources of injury, such as the presence of bacterial lipopolysaccharides (LPS) and an increase in pro-inflammatory cytokines, such as IL-7, NK cells, and T lymphocytes, thus determining a Th1 or Th2 immune response pattern [51,52,53].

MIP-1β, by exerting a chemoattractive effect on inflamed tissues in cells of the immune system, such as monocytes, macrophages, natural killer (NK) cells, and dendritic cells, mobilizes an increase in the circulating quantity of these cells and exacerbates the inflammatory response, causing damage to patients with CKD [51,52]. In this way, reducing MIP-1β using microcapsules with curcumin and propolis causes less activation of immune cells that trigger an inflammatory response, such as those mentioned above, thereby reducing the patient’s general inflammatory condition. MIP-1β activates G protein-coupled cell receptors, such as CCR. Directing cell migration from macrophages and polymorphonuclear leukocytes to chemokine-releasing cells modulates the response to induced stimuli [53].

Chang et al. (2023) [52] evaluated the potential of MIP-1 as a therapeutic target in diabetic renal protection (DKD). They observed that high levels of MIP-1β may play a role in the pathogenesis of DKD, as MIP-1β inhibition protects against podocyte damage, reduces glomerulosclerosis and fibrosis, and inhibits inflammation activation.

Due to its anti-inflammatory phenolic compounds, Brazilian propolis tends to reduce MIP-1β plasma levels in patients with CKD undergoing HD when supplemented at 400 mg/day for eight weeks [38].

We found no significant differences in the MDA levels after the intervention. When providing a dose of 1500 mg/day of turmeric for two months to patients undergoing HD, Pakfetrat et al. (2015) [23] found a significant reduction in MDA plasma levels. However, Rodrigues et al. (2021) [54] did not observe a significant difference in MDA levels in patients on HD supplemented with 1000 mg saffron for three months. This suggests that, in addition to the type of treatment, a higher dose of saffron may be necessary to reduce the plasma levels of MDA from its bioactive compounds.

In the present study, although *Curcuma longa* L. and green propolis demonstrated anti-inflammatory activities, no significant differences were observed in the plasma levels of uremic toxins (pCS, IS, and IAA).

The dosages used in our study may have been insufficient to induce substantial modulation of the gut microbiota, which plays a central role in toxin generation. Modifying the microbiota composition may require higher doses or longer durations of supplementation to achieve detectable changes in the microbial pathways involved in toxin generation. Salarolli et al. (2021) [55] showed a significant reduction in plasma pCS levels in patients undergoing hemodialysis after receiving 2.5 g of turmeric juice for three months. It is essential to highlight that both the dose and duration of supplementation in that study were substantially higher than those used in the present trial, which may partially explain the discrepancy in the outcomes related to uremic toxin levels. These findings reinforce the hypothesis that prolonged exposure to and/or higher doses of these bioactive compounds are necessary to effectively modulate gut microbiota activity and consequently reduce the production of gut-derived uremic toxins. An experimental model of CKD in rats that received 75 mg/kg curcumin for five weeks showed that the plasma levels of IS were not reduced [56]. In contrast, a study by our group showed that supplementation with 400 mg of green propolis extract per day for eight weeks in patients undergoing HD did not alter the gut microbiota composition or plasma levels of uremic toxins [57].

Combining bioactive compounds from different foods has been described as a way to enhance the beneficial effects of these compounds. The scientific literature still lacks sufficient evidence on this subject, making this unprecedented study of great relevance. As an example of the associations already made, we can mention Palliyage et al. (2021) [58], who, when they combined curcumin and resveratrol in nanoparticles, observed good stability of the compound, and the synergism of the formulation made the combination of curcumin and nano-encapsulated resveratrol more efficiently reduce the viability of cancer cells when compared to curcumin and resveratrol alone.

Abdolahi et al. (2017) [59] tested the effects of curcumin associated with omega-3 in humans with migraine. They observed a significantly greater reduction in the serum levels of TNF-α in the combined group compared to other groups, thus indicating the synergistic potential of the association.

The combination of propolis and turmeric used in this study was primarily based on the excellent results obtained by this research group in previous studies in which turmeric and propolis were used separately [38,60].

Curcumin has been described as being able to mitigate inflammation in CKD through several mechanisms. It stimulates the positive regulation of cytoprotective and antioxidant pathways, reduces mitochondrial damage by decreasing ROS production, and reacts directly by eliminating ROS and regulating the antioxidant pathways. In addition, it induces the master regulator of the antioxidant response, Nrf2, by modifying Keap1, which antagonizes the nuclear transcription factor factor-κB (NF-κB), an essential regulator of genes that encodes inflammatory cytokines during inflammatory responses, and also prevents their migration to the nucleus, as it inhibits its degradation of the IκB-binding complex [61]. Propolis, in turn, has been associated with several benefits, including the modulation of pro-inflammatory cytokines, such as IL-6 and MIP-1, as well as the reduction of adhesion molecules. Propolis can inhibit the endothelin and vascular endothelial growth factor and, like turmeric, acts to inhibit NF-κB; it is essential to mention that chrysin, a flavonoid with anti-inflammatory capacity, plays a vital role in this [38,62]. Thus, in future studies, we will continue to study these compounds to assess their effects.

The main limitation of this study was the COVID-19 pandemic, which not only restricted the collection of anthropometric data but also required a shorter intervention period to ensure participant safety and adherence, ultimately contributing to the feasibility of the trial. Additionally, it was not possible to apply longer questionnaires to assess the quality of life. Additionally, this study was likely underpowered to detect modest changes in some biomarkers, such as IL-6, TNF-α, and uremic toxins. The small sample size may have affected our ability to identify statistically significant differences between the groups. A crossover study is necessary to confirm that microcapsules containing propolis and curcumin may serve as anti-inflammatory agents in patients with CKD undergoing hemodialysis (HD).

## 5. Conclusions

Supplementation with 130 mg of curcuminoids and 500 mg of microencapsulated green propolis extract in patients with CKD undergoing hemodialysis for eight weeks demonstrated potential anti-inflammatory effects, as observed through the reductions in CRP and MIP-1. However, as a pilot study, these findings are preliminary and should be interpreted cautiously until replicated in larger, adequately powered clinical trials. Further studies are necessary to introduce nonpharmacological strategies based on bioactive compounds in food for patients with CKD, considering that they are already exposed to a high daily load of medication.

## Figures and Tables

**Table 1:** Chemical composition of the daily dose of microcapsules offered in the study.

Component	Fractions	(mg/Day)
Propolis	Total Flavonoids (Quercetin)	25.0
	Total phenolics (Gallic acid)	32.75
	Artepellin C	32.37
Turmeric	Curcumin	103.2
	Demethoxycurcumin	25.2
	Bisdemethoxycurcumin	5.76

**Table 2:** Baseline general and anthropometric characteristics of the patients on HD.

Parameters	Overall (N = 38)	Placebo (N = 20)	Microcapsules (N = 18)	*p*-Value
Sex (F/M)	20 (52.6%)/18 (47.4%)	10 (50%)/10 (50%)	10 (55.6%)/8 (44.4%)	0.986
Age (Years)	49 (17.7)	49 (18.7)	49 (16.2)	0.907
BMI (kg/m^2^)	23.03 (4.1)	24.34 (3.4)	21.6 (5.2)	0.034
Time on HD (Months)	35.0 (33)	31.5 (24.7)	37.5 (41.7)	0.527
Kt/V	1.5 (0.5)	1.4 (0.5)	1.6 (0.4)	0.096
HAS	27 (71.1%)	17 (85%)	10 (55.6%)	0.101
DM	6 (15.8%)	4 (20%)	2 (11.1)	0.761

Data are presented as median (interquartile range: IQR) or absolute (relative) proportions. *p*-values were obtained by using chi-squared tests (categorical nominal variables) or non-parametric Mann–Whitney U tests (numerical continuous variables). Abbreviations: HD: hemodialysis; Kt/V: dialysis adequacy; HAS: hypertension; DM: diabetes mellitus.

**Table 3:** Biochemical parameters of the groups at baseline.

Biochemical Parameters	Overall (N = 38)	Placebo (N = 20)	Microcapsules (N = 18)	*p*-Value
Total cholesterol (mg/dL)	143.5 (35.2)	138.0 (36.0)	145.5 (32.7)	0.682
High-density lipoprotein (mg/dL)	39.5 (14.7)	37 (11.5)	44 (12.3)	0.160
Low-density lipoprotein (mg/dL)	70.1 (24.4)	68.8 (24.1)	72.8 (18.6)	0.806
Albumin (mg/dL)	3.1 (0.4)	3.2 (0.4)	3.1 (0.3)	0.536
Phosphorus (mg/dL)	5.2 (2.3)	5.6 (2.3)	4.8 (2.2)	0.388
Calcium (mg/dL)	9.6 (1.2)	9.8 (1.2)	9.4 (1.0)	0.320
Triglycerides (mg/dL)	105.5 (83.2)	105.5 (96.0)	105.5 (64.2)	0.770
Urea (mg/dL)	107.5 (41.7)	102.5 (42.7)	109.5 (37.5)	0.292
Creatinine (mg/dL)	9.4 (3.6)	9.4 (3.5)	9.4 (3.6)	0.630
C-reactive protein (mg/dL)	4.1 (4.3)	2.8 (3.2)	4.7 (4.5)	0.105

Data are presented as median (interquartile range: IQR). *p*-values were obtained by using chi-squared tests (categorical nominal variables) or non-parametric Mann–Whitney U tests (numerical continuous variables).

**Table 4:** Dietary intake of patients with CKD undergoing hemodialysis.

Food Intake	Overall (N = 38)	Placebo (N = 20)	Microcapsules (N = 18)	*p*-Value
Energy (Kcal/d)	1352.4 (653.6)	1353 (500.9)	1350 (711.8)	0.988
Energy (Kcal/kg/d)	21 (12.1)	19.4 (8.3)	23.6 (14.5)	0.239
Proteins (g/d)	68.6 (42.8)	68.6 (36.3)	69.3 (44.6)	0.897
Proteins (g/kg/d)	1.1 (0.8)	1.0 (0.5)	1.1 (0.8)	0.421
Carbohydrates (g/d)	176.1 (79.7)	171.1 (54.7)	177.6 (98.6)	0.762
Lipids (g/d)	37.2 (27.2)	36.2 (31.2)	40.3 (25.7)	0.726
Monounsaturated (g/d)	12.6 (8.2)	12.1 (9.1)	13.3 (7.5)	0.988
Polyunsaturated (g/d)	6.2 (3.4)	6.5 (3.5)	5.2 (3.0)	0.520
Saturated (g/d)	14.7 (13.2)	14.1 (15.4)	16 (10.0)	1.000
Cholesterol (g/d)	275.3 (187.4)	277.8 (181.3)	275.3 (197.3)	0.828
Calcium (mg/d)	241.4 (225.8)	258.6 (194.3)	235.4 (287.9)	0.443
Iron (mg/d)	6.87 (4.9)	7.3 (2.4)	6.7 (3.0)	0.225
Fiber (g/d)	19.9 (8.2)	20.7 (11.3)	18.98 (7.2)	0.165
Sodium (mg/d)	1231.5 (924.5)	1240.7 (762.0)	1225 (1032.7)	0.919

Data are presented as median (interquartile range: IQR). *p*-values were obtained by using chi-squared tests (categorical nominal variables) or non-parametric Mann–Whitney U tests (numerical continuous variables).

**Table 5:** Baseline plasma levels of inflammatory cytokines, uremic toxins, and MDA in the placebo and microcapsules groups.

Parameters	Overall (N = 38)	Placebo (N = 20)	Microcapsules (N = 18)	*p*-Value
Cytokines				
IL-6 (pg/mL)	74.9 (79.1)	76.9 (101.0)	74.9 (30)	0.988
IL-8 (pg/mL)	5.3 (13.2)	4.5 (9.0)	6.1 (19.1)	0.678
MIP-1β (pg/mL)	42.8 (31.4)	46.5 (19.9)	32.9 (39.7)	0.678
GM-CSF (pg/mL)	0.2 (0.17)	0.2 (0.19)	0.2 (0.08)	0.865
TNF-α (pg/mL)	21.4 (37.7)	17.8 (15.4)	28.7 (44.7)	0.061
Uremic Toxins				
IS (mg/L)	15.0 (15)	12.6 (15.9)	17.9 (10.8)	0.141
pCS (mg/L)	24.2 (24.0)	21.8 (28.3)	25.2 (16.2)	0.438
IAA (ug/L)	1181.3 (670.1)	1254.8 (934.5)	1144.6 (406.9)	0.409
Lipid Peroxidation Marker				
MDA (mmol/L)	0.67 (1.3)	0.56 (2.1)	0.75 (1.1)	0.659

Data are presented as median (interquartile range: IQR). Abbreviations: IL: interleukin; MIP-1β: macrophage inflammatory protein-1β; GM-CSF: Granulocyte and Macrophage Colony-Stimulating Factor; TNF-α: tumor necrosis factor-alpha; G-CSF: Granulocyte colony-stimulating factor; MDA: malondialdehyde; IS: indoxyl sulfate; pCS: P-Cresyl sulfate; IAA: Indol acetic acid.

## Data Availability

The data presented in this study are available on request from the corresponding author. The data are not publicly available due to ethical restrictions involving participant confidentiality and the terms of informed consent, which do not include permission for public data sharing. Additionally, the dataset contains sensitive information that could potentially lead to the identification of individuals, even in anonymized form.

## Supplementary Materials

The following supporting information can be downloaded at: https://www.mdpi.com/article/10.3390/life15060891/s1: Figure S1. Chromatographic profile of Curcuma longa L and propolis capsules (1) caffeic acid; (2) p-coumaric; (3) 3.5-dicaffeoylquinic acid; (4) 4,5-dicaffeoylquinic acid; (5) aromadendrin; (6) drupanin; (7) artepillin C and (8) Baccarin. Figure S2. Chromatographic profile of Curcuma longa L. and propolis capsules (1) bidemethoxycurcumin; (2) demethoxycurcumin; (3) curcumin.
