# Supplementary material for: Effects of Curcuma longa L. and Green Propolis Extract-Loaded Microcapsules Supplementation on Inflammation in Hemodialysis Patients: Preliminary Results of a Randomized Clinical Trial
Source: Life (Basel). 2025 May 30;15(6):891. doi: 10.3390/life15060891 (PMC12194495; doi:10.3390/life15060891)
Supplement: Supplementary file 1 [file life-15-00891-s001.zip › life-3635812-supplementary.pdf]

life

MDPI

Article

Effects of *Curcuma longa L.* and green propolis extract loaded micro-capsules supplementation on inflammation in hemodialysis patients: Preliminary results of a Randomized Clinical Trial

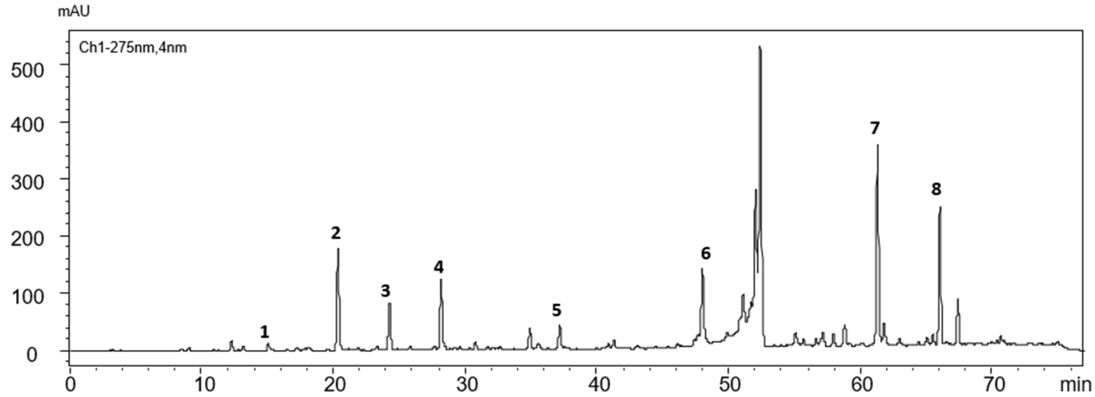

Figure S1. Chromatographic profile of *Curcuma longa L* and propolis capsules (1) caffeic acid; (2) p-coumaric; (3) 3,5-dicaffeoylquinic acid; (4) 4,5-dicaffeoylquinic acid; (5) aromadendrin; (6) drupanin; (7) artepillin C and (8) Baccarin.

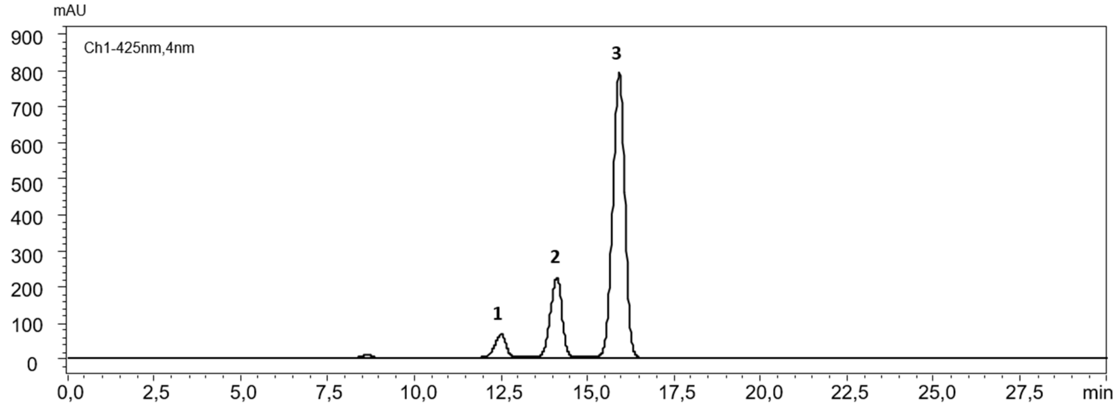

Figure S2. Chromatographic profile of *Curcuma longa L.* and propolis capsules (1) bidemethoxycurcumin; (2) demethoxycurcumin; (3) curcumin.
